# Updated Estimates of Childhood Diarrheal Morbidity in 11 Sub‐Saharan African Countries Using the Latest Demographic and Health Surveys (2021–2024): A Multilevel Modified Poisson Regression Analysis

**DOI:** 10.1155/bmri/2495286

**Published:** 2026-07-13

**Authors:** Temesgen Gebeyehu Wondmeneh

**Affiliations:** ^1^ Department of Public Health, College of Medical and Health Sciences, Samara University, Semera, Ethiopia, su.edu.et

**Keywords:** childhood diarrhea, Demographic and Health Surveys, diarrheal morbidity, multilevel modified Poisson regression, sub-Saharan Africa

## Abstract

**Background:**

Diarrheal disease remains a leading cause of morbidity among children under 5 years of age in sub‐Saharan Africa, yet many estimates rely on outdated data. Using the most recent Demographic and Health Survey (DHS) datasets, this study is aimed at providing updated estimates of childhood diarrheal morbidity and at identifying associated factors to inform monitoring and targeted interventions.

**Methods:**

This study analyzed pooled DHS data collected between 2021 and 2024 from 11 sub‐Saharan African countries, focusing on diarrheal morbidity among 124,167 children under 5 years of age. A multilevel modified Poisson regression model was employed to estimate adjusted prevalence ratios (APRs) and corresponding 95% confidence intervals (CIs), accounting for the hierarchical structure of the data, including clustering at household and community levels. Statistical significance was declared at *p* < 0.05.

**Results:**

The prevalence of diarrheal morbidity among children under 5 years of age was 13.8% (95% CI: 13.6%–14.0%), with significant variation across the included sub‐Saharan African countries. Lower prevalence was observed among children born to mothers aged 25–34 years (APR = 0.89; 95% CI: 0.85–0.92) and 35–49 years (APR = 0.82; 95% CI: 0.78–0.86), female children (APR = 0.93; 95% CI: 0.91–0.96), children aged 37–59 months (APR = 0.42; 95% CI: 0.40–0.44), children of literate mothers (APR = 0.86; 95% CI: 0.82–0.89), those from wealthier households (APR = 0.84; 95% CI: 0.79–0.88), children covered by health insurance (APR = 0.89; 95% CI: 0.83–0.95), and those who had received measles vaccination (APR = 0.95; 95% CI: 0.90–0.99). Conversely, distance to health facilities as a big problem (APR = 1.09; 95% CI: 1.05–1.13), underweight status (APR = 1.17; 95% CI: 1.10–1.24), and wasting (APR = 1.17; 95% CI: 1.09–1.26) were associated with a higher prevalence of diarrheal morbidity. Positive associations were also observed for media exposure, improved toilet facilities, four or more antenatal care visits, postnatal checkups, vitamin A supplementation, rotavirus vaccination, and deworming treatment. These associations should be interpreted cautiously, as they are unlikely to be causal and may reflect reverse causation, residual confounding, or reporting and detection bias.

**Conclusion:**

Childhood diarrhea remains a major public health concern in sub‐Saharan Africa, with substantial variation across the included countries. Diarrheal morbidity among children under 5 years of age was associated with a range of biological, socioeconomic, nutritional, and health service–related factors. Lower diarrheal morbidity was associated with older maternal age, female sex, older child age, maternal literacy, higher household wealth, health insurance coverage, and measles vaccination, whereas undernutrition and barriers to healthcare access were associated with higher morbidity. Counterintuitive associations observed for several health service and infrastructure variables likely reflect reverse causation and reporting bias rather than harmful effects. These findings underscore the need for integrated interventions aimed at improving child nutrition, reducing socioeconomic inequalities, and expanding equitable access to quality healthcare services.

## 1. Introduction

Diarrhea is a major public health problem, causing significant morbidity and mortality among children under five, especially in low‐resource countries with poor hygiene practices and limited access to treatment [1]. It is the third leading cause of death among children under five, responsible for approximately 443,832 deaths annually [2]. A meta‐analysis in Africa estimated that the prevalence of diarrhea among children under five was 23.6% [3]. Childhood diarrhea remains a major public health challenge and a leading cause of morbidity in sub‐Saharan Africa [4], with a reported prevalence of 18.4% among under‐five children in the region [5]. A recent synthesis indicated that nearly one quarter of children under five in East Africa experience diarrhea, highlighting the persistent vulnerability of young populations and the influence of household, environmental, and health service factors on disease occurrence [6].

Diarrheal diseases are driven by a complex interplay of determinants. Despite improvements in health services, nutrition, and sanitation, diarrhea continues to impose substantial health, economic, and developmental burdens on affected populations [7]. Nutritional status, including underweight and wasting, increases susceptibility by compromising immune defenses [8, 9]. Maternal and child factors, such as young child age and limited maternal education, have consistently been linked with higher diarrhea prevalence, highlighting the importance of caregiver knowledge and practices [6, 10]. A review in Africa further indicated that diarrhea was associated with young age, poor breastfeeding and hygiene, low socioeconomic status, and inadequate water and sanitation, while improved latrines and protected water sources were protective [3]. Higher household wealth and maternal age were protective against childhood diarrhea, whereas lack of improved water and sanitation increased the risk. Female children had a lower risk, while higher birth order [5] and prelacteal feeding [11] were associated with an increased risk.

A recent study by Jean Simon et al. [12] among Haitian mothers using Demographic and Health Survey (DHS) data highlighted the importance of contextual and behavioral determinants in childhood diarrheal disease and demonstrated that healthcare‐seeking behaviors are shaped by multiple intersecting factors, reinforcing the need for careful interpretation of cross‐sectional associations and consideration of health system interactions in diarrheal research.

Despite the substantial burden of childhood diarrhea in sub‐Saharan Africa, most existing estimates are based on earlier DHS rounds and do not reflect more recent national surveys conducted between 2021 and 2024. Moreover, there remains limited pooled, multicountry evidence that simultaneously accounts for hierarchical data structures and cross‐country variability. Country‐level differences in health systems; water, sanitation, and hygiene (WASH) infrastructure; and socioeconomic conditions may further contribute to heterogeneity in diarrhea prevalence and associated factors, yet they are not consistently accounted for in many prior studies.

From a methodological perspective, multilevel modified Poisson regression models are particularly appropriate for DHS data, as they account for the hierarchical structure of individuals nested within clusters and allow for the estimation of prevalence ratios for common outcomes, providing more interpretable effect measures than odds ratios. In addition, appropriate incorporation of survey weights, stratification, and clustering is essential to ensure valid and nationally representative estimates in pooled analyses.

Therefore, using the most recent DHS data from multiple sub‐Saharan African countries, this study is aimed at estimating the pooled prevalence of childhood diarrhea and identifying associated factors using a multilevel modeling approach. By integrating recent data and addressing both individual‐ and contextual‐level determinants, this study contributes updated, nationally representative, and policy‐relevant evidence to inform targeted interventions for reducing the burden of childhood diarrheal morbidity in sub‐Saharan Africa.

**Figure 1:**
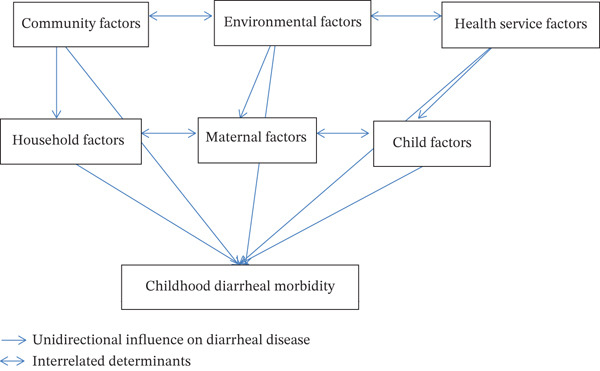
Conceptual framework of associated factors of childhood diarrheal morbidity.

Based on evidence from previous studies, a conceptual framework was developed to guide the selection and organization of explanatory variables. The framework illustrates the hypothesized relationships between child, maternal, household, environmental, health service, and community‐level factors and childhood diarrheal morbidity (Figure 1).

## 2. Methods

### 2.1. Study Design and Data Source

This study used data from the most recent (2021–2024) DHS, conducted in Burkina Faso, the Democratic Republic of the Congo (DRC), Côte d′Ivoire, Ghana, Kenya, Lesotho, Madagascar, Mali, Mozambique, Senegal, and Tanzania. DHS surveys are nationally representative, cross‐sectional household surveys implemented using standardized questionnaires, sampling procedures, and field protocols to ensure cross‐country comparability. DHS data, including survey questionnaires, methodology reports, and recode files, are accessible through the DHS Program website at https://www.dhsprogram.com/. The available microdata include core files such as the Children′s Recode (KR) file used in this analysis, which contains information on children aged 0–59 months and relevant health indicators. Access to DHS microdata requires free registration and approval of a data use request [13].

#### 2.1.1. Reporting Guideline

The reporting of this study conforms to the Strengthening the Reporting of Observational Studies in Epidemiology (STROBE) guideline for cross‐sectional studies (File S1) [14].

### 2.2. Study Population

The study population consisted of children aged 0–59 months residing in sampled households. Information on child health and household characteristics was obtained from mothers or primary caregivers and recorded in the KR dataset.

### 2.3. Inclusion and Exclusion Criteria

Children aged 0–59 months were included if they were usual household residents (or had slept in the household the night before the survey) and had complete data on diarrheal morbidity and key explanatory variables. Children who had died prior to the survey, those with “do not know” responses, or de jure nonresidents for key independent variables were excluded to avoid potential bias in effect estimates. Although including “do not know” responses in the denominator, as per DHS guidelines [15], had minimal impact on overall estimates, their exclusion ensured that only confirmed data were analyzed, minimizing misclassification and bias.

### 2.4. Sampling Design and Sample Size Determination

**Figure 2:**
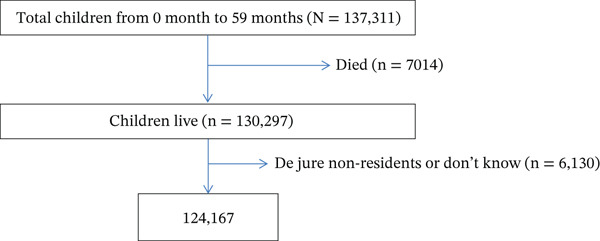
Flowchart of the selection procedure for study participants.

All DHS surveys employed a stratified, two‐stage cluster sampling design based on the most recent national population and housing censuses of each country. In the first stage, enumeration areas (EAs), also referred to as clusters, were selected independently within each stratum using probability proportional to size (PPS). Stratification was typically defined by administrative regions crossed with urban and rural residence, ensuring adequate representation across geographic and residential domains. In the second stage, a systematic random sample of households was selected from a complete household listing within each selected EA, and all eligible households were interviewed [13]. A total of 137,311 children aged 0–59 months were identified from the DHS KR file. After excluding children who had died prior to the survey (*n* = 7014) and those with “do not know” responses for diarrheal morbidity or who were de jure nonresidents for key independent variables (*n* = 6130), a total of 124,167 children remained for analysis. A sensitivity analysis including the “do not know” responses in the denominator, conducted in accordance with DHS recommendations [15], showed a slight reduction in diarrhea prevalence from 13.8% to 13.1%, indicating minimal impact on the overall estimates and supporting the validity of the final sample. In all analyses, the complex DHS survey design was explicitly incorporated through the use of sampling weights, stratification variables, and primary sampling units (clusters). This approach ensured nationally representative estimates and appropriate variance estimation for pooled analyses (Figure 2).

The analysis included children from 11 sub‐Saharan African countries, with a total weighted sample size of 115,836. The largest weighted samples were from the DRC (*n* = 18,468) and Kenya (*n* = 15,599), while Lesotho (*n* = 2046) had the smallest sample (Table 1).

**Table 1:** Number of children included in the analysis by country and total sample.

No.	Country	Unweighted sample size	Weighted sample size
1.	Burkina Faso	11,245	11,198
2.	DRC	21,759	18,468
3.	Côte d′Ivoire	9682	8773
4.	Ghana	8915	7711
5.	Kenya	18,613	15,599
6.	Lesotho	2233	2046
7.	Madagascar	11,318	11,665
8.	Mali	13,713	13,381
9.	Mozambique	7679	8431
10.	Senegal	8999	8596
11.	Tanzania	10,011	9968
12.	Total sample size	124,167	115,836

### 2.5. Variables and Operational Definitions

#### 2.5.1. Dependent Variable

##### 2.5.1.1. Diarrheal Morbidity

Diarrheal morbidity was defined according to DHS guidelines as the occurrence of diarrhea during the 2 weeks preceding the survey, based on maternal or caregiver report (DHS Variable H11). The outcome was coded as yes = 1 and no = 0. Children with “do not know” responses were excluded [15].

#### 2.5.2. Independent Variables

The selection of explanatory variables was guided by the conceptual framework presented in Figure 1, which was adapted from previous literature on childhood diarrheal morbidity. The framework considered factors operating at multiple levels, including child characteristics, maternal factors, household socioeconomic conditions, environmental sanitation factors, healthcare utilization indicators, and community‐level influences. This framework informed the selection of explanatory variables and the specification of the multilevel modified Poisson regression models. The variables included sociodemographic factors (place of residence, maternal age, marital status, employment status, maternal education, wealth index, religion, and maternal literacy), child characteristics (age, sex, birth order, birth interval, and number of under‐five children in the household), health service utilization indicators (measles vaccination, rotavirus vaccination, vitamin A supplementation, deworming, antenatal care [ANC] visits, postnatal check‐ups, health insurance coverage, and perceived distance to a health facility), media exposure, environmental factors (source of drinking water and type of sanitation facility), and nutritional status indicators (stunting, underweight, and wasting). Detailed variable categorizations are presented in Table 2.

**Table 2:** Independent variables.

Domain	Variable	Categories
Sociodemographic factors	Place of residence	Urban and rural
	Maternal age (years)	15–24, 25–34, and 35–49
	Marital status	Married (married or living with partner) and unmarried (never married, divorced, widowed, or separated)
	Employment status	Unemployed and employed
	Maternal education	No education, primary, and secondary and above
	Wealth index	Poor (poorest & poorer), middle, and rich (richer & richest)
	Religion	Christian, Muslim, animist, and no religion
	Maternal literacy	Literate (can read part or whole sentence) and illiterate
Child characteristics	Child age (months)	0–36 and 37–59
	Child sex	Male and female
	Birth order	First‐born and subsequent
	Birth interval (months)	6–23 and ≥ 24
	Number of under‐five children	≤ 2 and > 2
Health service utilization	Measles vaccination	Yes and no
	Rotavirus vaccination	Yes and no
	Vitamin A supplementation	Yes and no
	Deworming	Yes and no
	ANC visits	None, 1–3, and ≥ 4
	Postnatal check‐up	Yes and no
	Health insurance coverage	Yes and no
	Distance to health facility	Big problem and not a big problem
Media exposure	Media exposure	Exposed (radio, TV, or newspaper at least once per week) and not exposed
Environmental factors	Source of drinking water	Improved and unimproved
	Toilet sanitation facility	Improved and unimproved
Nutritional status	Stunting	HAZ < −2 SD and ≥ −2 SD
	Underweight	WAZ < −2 SD and ≥ −2 SD
	Wasting	WHZ < −2 SD and ≥ −2 SD

Abbreviations: HAZ, height‐for‐age *Z*‐score; WAZ, weight‐for‐age *Z*‐score; WHZ, weight‐for‐height *Z*‐score.

### 2.6. Data Management and Analysis

After obtaining permission from the DHS Program website, datasets from all included countries were downloaded and merged for analysis using Stata Version 17 [16]. All analyses accounted for the DHS complex survey design by applying sampling weights (v005 divided by 1,000,000) in accordance with DHS analytical guidelines [15]. To account for differences in population size and sample allocation across countries, DHS sampling weights were denormalized prior to pooling by multiplying the country‐specific sampling weight by the ratio of the country′s under‐five population to the survey sample size. The pooled weight was then normalized to maintain the overall sample size while preserving population representation across countries. Survey stratification and clustering variables were incorporated using Stata′s survey (svy) commands to account for the multistage sampling design and obtain valid standard errors and confidence intervals. All descriptive statistics presented in tables and figures are weighted estimates unless otherwise stated. Descriptive statistics were computed for childhood diarrheal morbidity and all independent variables. Childhood diarrheal morbidity was summarized as prevalence with 95% confidence intervals, whereas categorical variables were presented as frequencies and percentages.

Missing data patterns were explored using descriptive summaries and cross‐tabulations. Several variables exhibited substantial missingness, ranging from 7.11% for distance to a health facility to 44.5% for ANC visits, with the exception of sanitation facility, which had minimal missingness (1.88%). Given the potential for complete‐case analysis to introduce bias and reduce statistical power, missing values were addressed using multiple imputation by chained equations (MICE) under the assumption that data were missing at random. The imputation model included all variables used in the analysis to preserve the relationships among variables and enhance the plausibility of the missing at random assumption. Nine variables with incomplete observations, including sanitation facility, distance to a health facility, ANC visits, postnatal check‐up, measles vaccination, rotavirus vaccination, vitamin A supplementation, religion, and health insurance coverage, were imputed using appropriate conditional models according to variable type, including binary logistic, multinomial logistic, and ordinal logistic regression models. Twenty imputed datasets were generated with 200 iterations and a burn in period of 10 iterations to ensure convergence and stability [17]. Imputation diagnostics, including trace plots and comparisons of observed and imputed value distributions, were used to assess convergence and the adequacy of the imputation model. Sensitivity analyses comparing complete‐case and multiply imputed analyses were performed to evaluate the robustness of the findings. Final estimates were pooled using Rubin′s rules.

Because childhood diarrheal morbidity was a binary outcome with a relatively high prevalence, associations between explanatory variables and diarrheal morbidity were examined using a modified Poisson regression model within the generalized linear model framework [18]. This modeling approach was selected because it directly estimates adjusted prevalence ratios (APRs), which are generally preferred over odds ratios for cross‐sectional studies with common outcomes [19]. Compared with logistic regression, the modified Poisson approach avoids overestimation of effect sizes and provides more interpretable measures of association when outcomes are common [20].

Given the hierarchical structure of the DHS data, where children are nested within households and households within clusters (EAs), observations within the same cluster may be correlated. Therefore, a multilevel modeling approach was considered appropriate to account for within‐cluster dependence and cluster‐level variation. The need for multilevel analysis was assessed using the intraclass correlation coefficient (ICC) and likelihood ratio tests. Accordingly, a multilevel modified Poisson regression model was fitted with random effects specified at the household and EA levels. Robust sandwich variance estimators were applied to obtain valid standard errors and account for potential model misspecification [17]. Country was modeled as a fixed effect in the multilevel analysis to control for unobserved differences between countries. Thus, the estimated associations reflect pooled relationships across the included sub‐Saharan African countries rather than effects specific to individual countries. Variables with *p* values less than 0.20 in the bivariable analysis were entered into the multivariable model, and statistical significance was declared at *p* < 0.05. Given the large number of predictors included in the model, multicollinearity was assessed using variance inflation factors (VIFs). All VIF values were below 5, indicating that multicollinearity was not a concern. Highly correlated variables were excluded or combined where appropriate to ensure model stability. Results are presented as APRs with 95% confidence intervals.

Model fit for the multilevel modified Poisson regression was evaluated using deviance (−2 log‐likelihood) and Akaike information criterion (AIC). Lower values of deviance and AIC indicate better model fit. Likelihood ratio tests were also used to compare nested models and determine whether adding predictors significantly improved model fit. The degree of clustering and unexplained heterogeneity across clusters was quantified using several measures. The ICC estimates the proportion of total variance attributable to differences between clusters and is calculated as $\text{ICC}={\sigma^{2}}_{\text{cluster}/}\left({\sigma^{2}}_{\text{cluster}}+3.29\right)$ [21, 22]. The proportional change in variance (PCV) reflects the proportion of cluster‐level variance explained by adding predictors and is calculated as PCV = (Variance_null_ − Variance_model_)/Variance_null_ × 100*%* [21, 22]. The median rate ratio (MRR) quantifies cluster‐level heterogeneity on the rate ratio scale and is calculated as $\text{MRR}=\exp\;\left[0.67452\times \times {\sigma^{2}}_{\text{cluster}}\right]$, where 0.6745 is the 75th percentile of a standard normal distribution. The MRR represents the median relative change in the outcome between two individuals with identical covariates from different clusters [21, 22].

### 2.7. Ethical Considerations

The DHS Program obtained ethical approval from the relevant national ethics committees and the ICF Institutional Review Board. Written informed consent was obtained from all participants prior to data collection. This study used deidentified, publicly available DHS data, and no additional ethical approval from the author′s institution was required.

## 3. Results

### 3.1. Demographic Characteristics of Study Participants

The analysis included 124,167 under‐five children, corresponding to a weighted sample of 115,836 children from 11 sub‐Saharan African countries. Most mothers were aged 25–34 years (45.9%). Children aged 0–36 months accounted for nearly two‐thirds of the sample (62.2%), and the sex distribution of children was almost equal, with a slight predominance of males (50.7%). The majority of mothers were married (86.4%) and employed (57.5%). Muslims constituted the largest religious group (47.2%), followed by Christians (35.0%). Most respondents resided in rural areas (69.5%). Over one‐third of mothers had secondary education or above (35.8%), while 34.2% had no formal education; however, a higher proportion of mothers were illiterate (59.6%). Nearly half of households were classified as poor (44.1%), and two‐thirds of mothers reported media exposure (66.7%). Most households had two or fewer under‐five children (72.3%), and the majority of children were of higher birth order (76.0%). About 60.3% of households used improved water sources, while access to improved sanitation was nearly evenly split, with 50.8% having improved toilet facilities. All descriptive statistics presented are weighted estimates (Table 3).

**Table 3:** Demographic characteristics of study participants.

Variables	Category	Weighted frequency	Percentages
Maternal age (in years)	15–24	34,416	29.71%
	25–34	53,158	45.89%
	35–49	28,262	24.4%
Children′s age	0–36 months	72,032	62.18%
	37–59 months	43,804	37.82%
Children′s sex	Males	58,692	50.67%
	Females	57,144	49.33%
Marital status	Unmarried	15,765	13.61%
	Married	100,071	86.39%
Employment status	Unemployed	49,290	42.55%
	Employed	66,546	57.45%
Religious	Muslim	47,186	47.21%
	Christian	34,952	34.97%
	Animist	7283	7.29%
	No religious	10,538	10.54%
Resident	Urban	35,367	30.53%
	Rural	80,469	69.47%
Mothers′ educational status	No education	39,592	34.18%
	Primary	34,793	30.04%
	Secondary and above	41,451	35.78%
Literacy	Illiterates	69,017	59.58%
	Literates	46,819	40.42%
Wealth index	Poor	51,092	44.11%
	Middle	22,692	19.59%
	Rich	42,052	36.3%
Media exposed	No	38,613	33.33%
	Yes	77,223	66.67%
Number of under‐five children	Less than or equal to two	83,760	72.31%
	Greater than two	32,076	27.69%
Birth orders	First	27,793	23.99%
	Subsequent	88,043	76.01%
Water source	Unimproved	46,000	39.71%
	Improved	69,836	60.29%
Types of toilet	Unimproved	55,931	49.21%
	Improved	57,719	50.79%

### 3.2. Nutritional Status and Health Service Utilization Characteristics

Among children, 9.3% were underweight, 15.9% were stunted, and 3.8% were wasted. The majority had a birth interval of 24 months or more (86.7%), whereas 13.3% were born with intervals shorter than 24 months. Health insurance coverage was low, with only 10.9% of households reporting coverage. Nearly two‐thirds of mothers attended four or more ANC visits (62.6%), while 7.8% had no ANC follow‐up. Postnatal checkups were reported by just over one‐quarter of mothers (27.1%). About 35.7% of respondents indicated that distance to a health facility was a big problem. Coverage of child health interventions varied: 54.6% of children were vaccinated against measles, 67.9% received rotavirus vaccination, and 55.3% received vitamin A supplementation. Less than half of the children had received deworming services (41.7%) (Table 4).

**Table 4:** Nutritional status and health service utilization characteristics.

Variables	Category	Weighted frequency	Percentages
Underweight	No	105,085	90.72%
	Yes	10,751	9.28%
Stunted	No	97,421	84.1%
	Yes	18,415	15.9%
Wasted	No	111,466	96.23%
	Yes	4370	3.77%
Birth interval	Below 24 months	15,418	13.31%
	24 and above months	100,418	86.69%
Health insurance coverage	No	89,366	89.15%
	Yes	10,871	10.85%
ANC visits	None	5066	7.83%
	1–3	19,137	29.59%
	4+	40,478	62.58%
Postnatal checkup	No	47,621	72.87%
	Yes	17,726	27.13%
Distance to health facility	Not a big problem	69,612	64.26%
	Big problem	38,709	35.74%
Measles vaccinated	No	31,402	45.41%
	Yes	37,752	54.59%
Rotavirus vaccinated	No	22,174	32.09%
	Yes	46,932	67.91%
Vitamin A supplementation	No	31,017	44.67%
	Yes	38,415	55.33%
Deworming	No	67,489	58.26%
	Yes	48,347	41.74%

### 3.3. Prevalence of Diarrheal Morbidity Among Under‐Five Children

**Figure 3:**
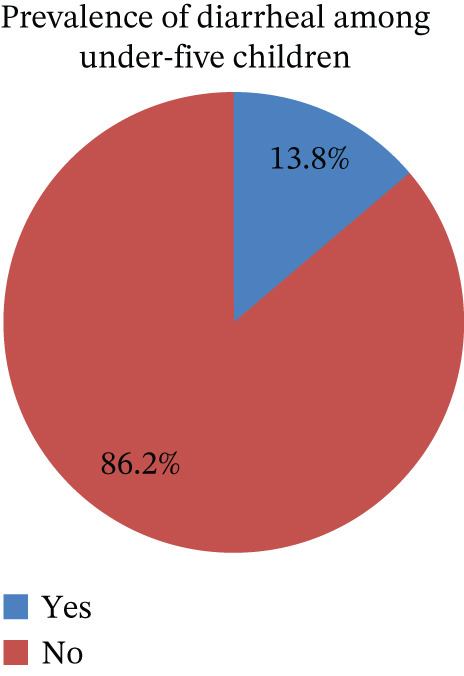
Prevalence of diarrheal morbidity among under‐five children in sub‐Saharan Africa.

**Figure 4:**
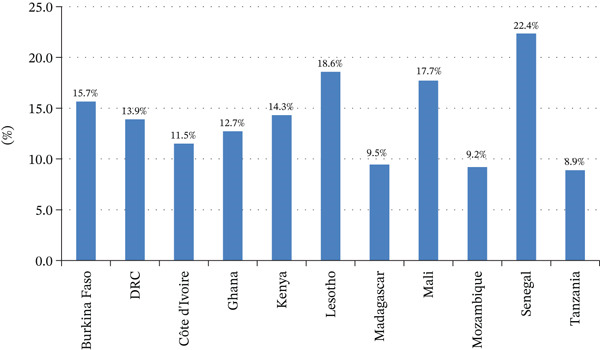
Prevalence of diarrhea among under‐five children by sub‐Saharan African country.

The prevalence of diarrheal morbidity among under‐five children in sub‐Saharan Africa was 13.8% (95% CI: 13.6%–14.0%) (Figure 3). Diarrhea prevalence among under‐five children varies widely, ranging from 8.9% in Tanzania to 22.4% in Senegal. The highest prevalence was observed in Senegal (22.4%), followed by Lesotho (18.6%) and Mali (17.7%). Diarrhea prevalence was 15.7% in Burkina Faso, 14.3% in Kenya, 13.9% in the DRC, 12.7% in Ghana, and 11.5% in Côte d′Ivoire. In contrast, lower prevalence was observed in Madagascar (9.5%), Mozambique (9.2%), and Tanzania (8.9%) (Figure 4).

### 3.4. Extent of Missing Data and Imputation Results

Among the 124,167 observations analyzed, several variables exhibited substantial missingness before imputation. The highest proportions of missing data were observed for ANC visits (44.5%), postnatal check‐ups (44.0%), and child health variables, including measles vaccination (40.2%), rotavirus vaccination (40.2%), and vitamin A supplementation (40.0%). Moderate levels of missingness were found for religion (16.4%), health insurance coverage (15.0%), and distance to a health facility (7.11%), whereas the sanitation facility had minimal missingness (1.88%).

Diagnostic assessments indicated satisfactory convergence and stability of the imputation process across the 20 datasets. The distributions of imputed values were comparable to those of the observed data, supporting the plausibility of the imputed values. Furthermore, sensitivity analyses comparing complete case and multiply imputed analyses yielded similar effect estimates, suggesting that the study findings were robust to the handling of missing data. Therefore, the multiply imputed datasets were used for all subsequent analyses.

### 3.5. Sensitivity Analysis

A sensitivity analysis was conducted by comparing complete‐case results (Table S1) with multiple imputation estimates (Table 5). Overall, results were consistent across both approaches, showing similar directions and magnitudes for most associations. Maternal age, household wealth, literacy, media exposure, health insurance, postnatal checkup, child age, child sex, underweight, wasting, vitamin A supplementation, rotavirus vaccination, deworming, and country of residence remained significantly associated with childhood diarrhea in both analyses. Marital status, employment status, ANC attendance, water source, stunting, and number of under‐five children in the household were consistently nonsignificant. Some differences were observed: Maternal education, birth order, and birth interval were significant in the complete‐case analysis but not after imputation, while measles vaccination became significant only in the imputed model. Overall, the findings suggest robustness to missing data. However, changes in significance for a few variables indicate that complete‐case analysis may have reduced precision and potentially introduced bias due to missing data exclusion. Details of complete‐case analysis results are provided in Table S1.

**Table 5:** Factors associated with diarrhea among under‐five children.

Variable	Category	Null model	Individual level	Community level	Individual and community level
			APR (95% CI)	APR (95% CI)	APR (95% CI)
Maternal age (in years)	15–24		1		1
	25–34		0.89 (0.86–0.92)		**0.89 (0.85–0.92)**
	35–49		0.82 (0.78–0.86)		**0.82 (0.78–0.86)**
Maternal education	No education		1		1
	Primary		1.04 (0.99–1.09)		1.04 (1.0–1.09)
	Secondary and above		1.04 (0.98–1.09)		1.04 (0.99–1.1)
Wealth index	Poor		1		1
	Middle		0.97 (0.93–1.01)		0.96 (0.92–1.0)
	Rich		0.87 (0.83–0.91)		**0.84 (0.79–0.88)**
Marital status	Unmarried		1		1
	Married		1.01 (0.96–1.06)		1.01 (0.97–1.06)
Employment status of mothers	Unemployed		1		1
	Employed		1.027 (0.99–1.06)		1.03 (1.0–1.07)
Religion	Muslim		1		1
	Christian		0.87 (0.84–0.91)		**0.87 (0.84–0.91)**
	Animist		0.77 (0.71–0.84)		**0.77 (0.71–0.84)**
	No religion		1.01 (0.95–1.08)		1.01 (0.94–1.07)
Residence	Urban			1	1
	Rural			0.94 (0.9–0.99)	**0.94 (0.9–0.99)**
Literacy	Illiterates		1		1
	Literates		0.85 (0.82–0.89)		**0.85 (0.82–0.89)**
Media exposure	Unexposed		1		1
	Exposed		1.12 (1.08–1.16)		**1.12 (1.08–1.16)**
Health insurance	No		1		1
	Yes		0.89 (0.84–0.95)		**0.89 (0.84–0.95)**
ANC visits	No ANC		1		1
	1–3		1.05 (0.97–1.12)		1.05 (0.98–1.13)
	4 and above		1.13 (1.05–1.21)		**1.13 (1.05–1.21)**
Postnatal checkup	No		1		1
	Yes		1.12 (1.08–1.16)		**1.12 (1.08–1.16)**
Health facility distance	No big problem			1	1
	Big problem			1.07 (1.04–1.1)	**1.07 (1.04–1.1)**
Child age (in months)	0–36		1		1
	37–59		0.42 (0.41–0.44)		**0.42 (0.4–0.44)**
Child sex	Males		1		1
	Females		0.93 (0.91–0.96)		**0.93 (0.91–0.96)**
Birth order	First		1		1
	Subsequent		1.01 (0.97–1.05)		1.01 (0.97–1.05)
Birth interval (in months)	≤ 24		1		1
	> 24		0.98 (0.94–1.03)		0.99 (0.95–1.03)
Number of under‐5 children	≤ 2		1		1
	≥ 3		1.02 (0.98–1.06)		1.02 (0.99–1.06)
Water source	Unimproved		1		1
	Improved		1.0 (0.96–1.04)		0.99 (0.96–1.03)
Toilet type	Unimproved		1		1
	Improved		1.06 (1.02–1.1)		**1.06 (1.02–1.10)**
Underweight	No		1		1
	Yes		1.17 (1.1–1.24)		**1.17 (1.1–1.24)**
Stunted	No		1		1
	Yes		0.99 (0.95–1.04)		0.99 (0.95–1.04)
Wasted	No		1		1
	Yes		1.17 (1.09–1.26)		**1.17 (1.09–1.26)**
Measles vaccine	No		1		1
	Yes		0.94 (0.9–0.99)		**0.95 (0.9–0.99)**
Vitamin A	No		1		1
	Yes		1.18 (1.13–1.22)		**1.18 (1.13–1.22)**
Rotavirus	No		1		1
	Yes		1.2 (1.15–1.25)		**1.2 (1.15–1.25)**
Deworming	No		1		1
	Yes		1.098 (1.058–1.14)		**1.1 (1.06–1.14)**
Country	Burkina Faso			1	1
	DR Congo			0.95 (0.88–1.02)	0.97 (0.89–1.05)
	Cote d′Ivoire			0.68 (0.62–0.75)	**0.67 (0.61–0.74)**
	Ghana			0.87 (0.8–0.96)	0.91 (0.8–1.03)
	Kenya			0.92 (0.86–0 0.99)	**0.86 (0.79–0.93)**
	Lesotho			1.14 (1.02–1.3)	0.98 (0.87–1.1)
	Madagascar			0.64 (0.59–0.7)	**0.6 (0.55–0.66)**
	Mali			1.11 (1.02–1.2)	**1.1 (1.01–1.2)**
	Mozambique			0.71(0.64–0.79)	**0.68 (0.61–0.76)**
	Senegal			1.33 (1.22–1.45)	**1.34 (1.23–1.47)**
	Tanzania			0.55 (0.5–0.61)	**0.52 (0.47–0.58)**
Model fitness diagnosed					
Deviance		101,760.72	98,515.98	101,736.86	98,465.64
AIC		101,764.7	98,581.99	101,744.9	98,535.65
BIC		101,784.2	98,903.06	101,783.8	98,876.18
Measure of variation					
ICC		1.388%	1.1916%	1.387%	1.1894%
MRR		1.2269	1.2096	1.2268	1.2088
PCV		—	13.68%	0.022%	13.83%

*Note:* Statistically significant associations (*p* < 0.05 and 95% confidence intervals that do not include 1) are presented in bold.

Abbreviations: 95% CI, 95% confidence interval; AIC, Akaike information criterion; ANC, antenatal care; APR, adjusted prevalence ratio; BIC, Bayesian information criterion; ICC, intraclass correlation coefficient; MRR, median rate ratio; PCV, proportional change in variance.

### 3.6. Model Fitness

The null model showed the poorest fit, with the highest deviance (101,760.72), AIC (AIC = 101,764.7), and Bayesian information criterion (BIC) (BIC = 101,784.2), indicating substantial unexplained community‐level variability in childhood diarrhea across sub‐Saharan Africa. Inclusion of individual‐level factors resulted in a marked improvement in model fit, with a substantial reduction in deviance (98,515.98) and corresponding decreases in AIC (98,581.99) and BIC (98,903.06). This improvement suggests that individual‐level characteristics explain a considerable proportion of the variability in childhood diarrhea. In contrast, the model including only community‐level factors showed minimal improvement over the null model (deviance = 101,736.86; AIC = 101,744.90; and BIC = 101,783.80), indicating that the measured community‐level variables alone had limited explanatory power. The full model, which incorporated both individual‐ and community‐level factors, demonstrated the best overall fit, with the lowest deviance (98,465.64), AIC (98,535.65), and BIC (98,876.18). This indicates that jointly modeling individual and community characteristics provides the most parsimonious and well‐fitting explanation of diarrheal morbidity among under‐five children, even after accounting for model complexity (Table 5).

### 3.7. Measure of Variation

The ICC declined slightly from 1.39% in the null model to 1.19% in the full model, showing that although most variation in childhood diarrhea occurs at the individual level, a small but meaningful proportion remains attributable to community‐level differences. The MRR also decreased from 1.23 in the null model to 1.21 in the fully adjusted model. In the fully adjusted model, the MRR of 1.21 indicates that, for two children with similar characteristics, the median difference in diarrhea rates between a higher risk and a lower risk community was approximately 21%, reflecting persistent unobserved community‐level heterogeneity. The PCV further showed that individual‐level factors explained 13.68% of the between‐community variance, whereas community‐level factors alone explained almost none (0.02%). The full model explained 13.83% of the variance, indicating that most of the explained heterogeneity in diarrheal disease risk is driven by individual‐level determinants, with limited contribution from the measured community‐level characteristics (Table 5).

### 3.8. Assessment of Multicollinearity

VIFs were assessed to check for multicollinearity among the independent variables. VIF values ranged from 1.00 to 1.92, with a mean of 1.35, all well below the commonly used threshold of 5. This indicates that multicollinearity was not a concern, and all predictors were retained in the final model.

### 3.9. Factors Associated With Diarrhea Among Under‐Five Children

Children born to older mothers had a lower prevalence of diarrhea compared with those born to younger mothers, including mothers aged 25–34 years (APR = 0.89; 95% CI: 0.85–0.92) and 35–49 years (APR = 0.82; 95% CI: 0.78–0.86). Female children were less likely to experience diarrhea than male children (APR = 0.93; 95% CI: 0.91–0.96). Increasing child age was strongly protective, with children aged 37–59 months exhibiting substantially lower diarrhea prevalence compared with younger children (APR = 0.42; 95% CI: 0.40–0.44). Children of literate mothers had a lower prevalence of diarrhea (APR = 0.85; 95% CI: 0.82–0.89). Residence in rural areas was also associated with a reduced prevalence of diarrhea (APR = 0.94; 95% CI: 0.9–0.99). Children from Christian (APR = 0.87; 95% CI: 0.84–0.91) and animist households (APR = 0.77; 95% CI: 0.71–0.84) had a lower prevalence of diarrhea compared with the reference religious group. Children from rich households had a lower prevalence of diarrhea compared with those from poor households (APR = 0.84; 95% CI: 0.79–0.88). Distance to a health facility as a major problem was associated with an increased prevalence of diarrhea (APR = 1.07; 95% CI: 1.04–1.1). Unexpectedly, children from households with media exposure had a higher prevalence of diarrhea (APR = 1.12; 95% CI: 1.08–1.16). Similarly, children living in households with improved toilet facilities had a statistically significantly higher prevalence of diarrhea compared with those using unimproved toilets (APR = 1.06; 95% CI: 1.02–1.10). Underweight children had a higher prevalence of diarrhea (APR = 1.17; 95% CI: 1.10–1.24), as did wasted children (APR = 1.17; 95% CI: 1.09–1.26), compared with their well‐nourished counterparts. Children covered by health insurance had a lower prevalence of diarrhea (APR = 0.89; 95% CI: 0.84–0.95). In contrast, children whose mothers had four or more ANC visits experienced a higher prevalence of diarrhea (APR = 1.13; 95% CI: 1.05–1.21), as did children who received postnatal checkups (APR = 1.12; 95% CI: 1.08–1.16). Vitamin A supplementation (APR = 1.18; 95% CI: 1.13–1.22), rotavirus vaccination (APR = 1.20; 95% CI: 1.15–1.25), and deworming treatment (APR = 1.10; 95% CI: 1.06–1.14) were associated with higher reported diarrheal morbidity, whereas measles vaccination was associated with lower diarrheal morbidity (APR = 0.95; 95% CI: 0.90–0.99). Relative to Burkina Faso, under‐five children in Côte d′Ivoire, Kenya, Madagascar, Mozambique, and Tanzania exhibited lower prevalence ratios of diarrhea, whereas those in Mali and Senegal exhibited higher prevalence ratios (Table 5).

## 4. Discussion

This study estimated the prevalence of childhood diarrhea in sub‐Saharan Africa and examined its associated factors using pooled DHS data from 11 countries (2021–2024). The study contributes updated, comparable evidence on diarrheal morbidity using recent nationally representative datasets across multiple sub‐Saharan African countries.

The prevalence of diarrhea among children under five was 13.8% (95% CI: 13.6%–14.0%), which is lower than previous regional estimates, including a meta‐analysis in Africa reporting a prevalence of 23.6% [3], earlier reports from sub‐Saharan Africa indicating 18.4% among under‐five children [5], and estimates from East Africa [6]. This difference may be attributed to variations in survey periods, as well as improvements in maternal education and empowerment, socioeconomic conditions, access to preventive strategies, and health service utilization, which may have strengthened child‐care practices and reduced susceptibility to diarrhea [1, 3, 5, 10]. Despite the lower magnitude, diarrhea remains a significant public health concern, consistent with evidence that it is a leading cause of morbidity and mortality in this age group [1, 2, 4]. The persistent burden, even at 13.8%, underscores the continued vulnerability of young populations and reflects the complex interplay of biological, socioeconomic, nutritional, and environmental factors identified in prior studies [1–7].

Although the present study focused on estimating the pooled regional prevalence and associated factors, substantial variation in diarrhea prevalence was observed across the 11 countries included in the analysis, ranging from 8.9% in Tanzania to 22.4% in Senegal. These differences may reflect variations in socioeconomic development, nutritional status, healthcare access, environmental conditions, and the implementation and effectiveness of public health programs [1, 7–9]. Although country was included as a fixed‐effect covariate in the multilevel analysis to account for differences between countries, the persistence of substantial heterogeneity in diarrhea prevalence across countries suggests that important contextual factors may continue to influence childhood diarrheal morbidity. This finding underscores the need for country‐specific prevention and control strategies that address local determinants of disease.

Older maternal age was associated with lower diarrheal morbidity, consistent with previous studies [5], possibly reflecting greater caregiving experience and improved child‐care practices [6, 10]. Females and older children were less likely to experience diarrhea, in line with evidence that biological susceptibility and immune maturation influence infection risk [23]. Maternal literacy also showed a protective association, reflecting the importance of caregiver knowledge in recognizing early illness, adhering to recommended hygiene and nutrition practices, and seeking timely medical care [6, 7, 10]. These findings support previous evidence demonstrating that maternal education and empowerment contribute to improved child health outcomes in low‐resource settings [24].

Children from rich households had lower diarrheal morbidity, reflecting better living conditions, food security, and access to healthcare [3, 5]. Rural residence was associated with lower reported diarrhea prevalence, potentially due to lower population density and reduced exposure to overcrowding [5, 25]. Religious affiliation appeared protective in some groups, possibly reflecting behavioral or cultural practices [11] influencing hygiene and care‐seeking.

Underweight and wasted children had a higher prevalence of diarrhea, consistent with the bidirectional relationship between malnutrition and enteric infections. This finding aligns with previous evidence showing that acute malnutrition increases susceptibility to infection by compromising immune defenses [8, 9]. In contrast, stunting was not associated with diarrhea, likely because its chronic nature does not temporally correspond with the recent diarrheal episodes captured in cross‐sectional surveys. These results underscore the critical need to address acute malnutrition in efforts to reduce diarrhea‐related morbidity.

Improved sanitation was unexpectedly associated with higher reported diarrheal morbidity, whereas improved drinking water sources showed no significant association with diarrheal morbidity. This finding contrasts with those of previous studies [3, 5, 7]. Although improved sanitation is generally expected to be protective, the observed association may reflect noncausal explanations, given the cross‐sectional nature of the study, including shared sanitation facilities, inadequate maintenance, residual confounding, or differential health‐seeking and reporting behaviors rather than a true increase in risk. Furthermore, households with improved facilities may have greater health awareness and better recognition and reporting of childhood illnesses. Therefore, the findings underscore that access to infrastructure alone may be insufficient and that improvements in hygiene behavior, safe water handling, and sanitation practices are equally important components of effective WASH interventions.

Health service utilization and preventive interventions were also positively associated with reported diarrheal morbidity, including ANC, postnatal checkups, vitamin A supplementation, rotavirus vaccination, and deworming. These findings are inconsistent with those of previous studies [26, 27]. However, these associations are unlikely to represent causal effects and may instead reflect reverse causation, whereby children who experience diarrhea are more likely to come into contact with healthcare services. Increased interactions with health facilities may also contribute to surveillance and reporting bias, as caregivers who frequently access healthcare services may be more likely to recognize and report diarrheal episodes [28]. In addition, some preventive interventions may be delivered opportunistically during healthcare visits following illness episodes, which could further explain the observed associations. This interpretation is supported by the study of Jean Simon et al. [12], which demonstrated that healthcare‐seeking behavior for childhood diarrhea is influenced by multiple socioeconomic, behavioral, and contextual factors. Therefore, the observed associations should not be interpreted as evidence that these interventions increase diarrheal morbidity. Rather, they highlight the complex relationship between healthcare utilization, illness recognition, and reporting, underscoring the need for cautious interpretation of findings derived from cross‐sectional studies.

In contrast, measles vaccination was associated with lower diarrheal morbidity, consistent with previous evidence demonstrating that immunization reduces severe infectious diseases and secondary diarrheal complications [29].

### 4.1. Community‐Level Variability and Justification for Multilevel Modeling

Although the ICC was relatively low, statistically meaningful clustering of childhood diarrheal morbidity remained evident across communities. The MRR further indicated persistent between‐community heterogeneity after accounting for measured covariates. In addition, the fully adjusted model demonstrated superior fit compared with the null and partially adjusted models, as evidenced by lower deviance, AIC, and BIC values. These findings support the use of multilevel modified Poisson regression, which appropriately accounts for the hierarchical structure of DHS data and provides more reliable estimates than conventional single‐level models. Moreover, considering both individual‐ and community‐level characteristics allows a more comprehensive understanding of childhood diarrheal morbidity in sub‐Saharan Africa, even when the proportion of variance attributable to community‐level factors is relatively modest.

### 4.2. Policy Implications of the Findings

The findings have important implications for public health policy and practice in sub‐Saharan Africa. Interventions aimed at reducing childhood diarrheal morbidity should adopt a multisectoral approach that simultaneously addresses nutritional deficiencies, socioeconomic inequalities, maternal literacy, and barriers to healthcare access. Strengthening integrated WASH programs, improving maternal education, and expanding equitable access to child health services remain essential priorities. Given the substantial variation in diarrhea prevalence across countries, interventions should be adapted to local contexts rather than relying solely on region‐wide strategies. In addition, the unexpected associations observed with healthcare utilization and preventive interventions highlight the importance of strengthening surveillance systems and conducting longitudinal studies to better understand temporal relationships and minimize the influence of reverse causation and reporting bias.

### 4.3. Strengths and Limitations of the Study

This study has several strengths. First, it utilized pooled DHS data from 11 sub‐Saharan African countries collected between 2021 and 2024, providing recent and nationally representative estimates of childhood diarrheal morbidity. The large sample size improved statistical power and precision. Standardized DHS methodologies enhanced comparability across countries and strengthened external validity. Second, the use of multilevel modified Poisson regression appropriately accounted for the hierarchical nature of the data and yielded APRs, which are more interpretable than odds ratios for common outcomes. Third, missing data were addressed using multiple imputation by chained equations. Sensitivity analyses demonstrated that excluding “do not know” responses had minimal influence on prevalence estimates, supporting the robustness of the findings.

Despite these strengths, some limitations should be considered. First, the cross‐sectional nature of the DHS precludes causal inference, and all observed relationships should be interpreted as associations rather than causal effects. Reverse causality may be particularly relevant for health service utilization variables and preventive interventions. Second, diarrheal morbidity was based on maternal recall of symptoms during the 2 weeks preceding the survey and is, therefore, susceptible to recall bias and misclassification. Differential reporting according to socioeconomic or educational status may also have affected the estimates. Third, although variation in diarrhea prevalence was observed across the included countries, this heterogeneity was explicitly accounted for by including country fixed effects in the analysis. While pooled analyses enhance regional generalizability, the inclusion of country fixed effects adjusts for unobserved country‐level contextual differences that may influence the observed associations. Therefore, the findings represent adjusted regional‐average estimates across sub‐Saharan Africa rather than unadjusted pooled estimates that ignore country heterogeneity. Fourth, water and sanitation variables were based on facility classifications rather than direct measurements of water quality, hygiene practices, or environmental contamination, potentially leading to exposure misclassification. Fifth, several variables, including ANC, postnatal care, vaccination status, and vitamin A supplementation, had substantial proportions of missing observations. Although multiple imputation was employed under the assumption that data were missing at random, this assumption cannot be fully verified, and some degree of uncertainty may remain. Sixth, residual confounding from unmeasured factors such as seasonality, food hygiene, local disease outbreaks, environmental contamination, and cultural practices may have influenced the observed associations. Finally, although pooling data across countries enhanced generalizability, important country‐specific contextual differences may have been masked. Consequently, the results should be interpreted as regional estimates rather than country‐level effects. Future longitudinal and country‐specific studies are warranted to better understand causal pathways and contextual determinants of childhood diarrheal morbidity in sub‐Saharan Africa.

## 5. Conclusion

Childhood diarrhea remains an important public health challenge in the 11 sub‐Saharan African countries, with marked between‐country variation; relative to Burkina Faso, prevalence ratios were lower in several countries and higher in a few others. Diarrheal morbidity was associated with a complex interplay of child, maternal, household, nutritional, environmental, and healthcare‐related factors. Lower diarrheal morbidity was observed among children of older mothers, female children, older children, children of literate mothers, those from wealthier households, those covered by health insurance, and those who had received measles vaccination. Conversely, undernutrition and limited access to health facilities were associated with higher diarrheal morbidity. Some unexpected positive associations involving improved sanitation facilities, ANC visits, postnatal checkups, vitamin A supplementation, rotavirus vaccination, and deworming should be interpreted cautiously. These findings are unlikely to represent harmful effects and may instead reflect reverse causality, differential healthcare‐seeking behavior, reporting bias, residual confounding, and other limitations inherent to cross‐sectional studies. The findings underscore the importance of integrated and context‐specific strategies that address nutritional vulnerability, socioeconomic inequalities, maternal literacy, and equitable access to quality health services and effective WASH interventions. Given the considerable heterogeneity observed across countries, interventions should be tailored to local contexts rather than relying solely on region‐wide approaches. Future longitudinal and intervention‐based studies are warranted to clarify temporal relationships, better understand country‐specific determinants, and provide stronger evidence regarding the effects of sanitation and child health services on childhood diarrheal morbidity.

**Nomenclature**
DHS: Demographic and Health Survey
EA: enumeration area
DRC: Democratic Republic of the Congo
STROBE: Strengthening the Reporting of Observational Studies in Epidemiology
ANC: antenatal care
VIP: ventilated improved pit
SD: standard deviation
MICE: multiple imputation by chained equations
AIC: Akaike information criterion
BIC: Bayesian information criterion
ICC: intraclass correlation coefficient
MRR: median rate ratio
PCV: proportional change in variance
APR: adjusted prevalence ratio
CI: confidence interval

## Author Contributions

T.G.W. conceived the idea, extracted the data, performed the data analysis, and drafted the manuscript. He interpreted the analysis and revised the manuscript.

## Funding

No funding was received for this manuscript.

## Disclosure

The author has read and approved the final version of the manuscript.

## Ethics Statement

As this study involved secondary analysis of DHS data, obtaining informed consent from participants was not applicable. Instead, permission to access the data was granted by DHS International. All data were fully anonymized prior to access, in accordance with DHS International′s informed data use policies.

## Consent

The author has nothing to report.

## Conflicts of Interest

The author declares no conflicts of interest.

## Supporting Information

Additional supporting information can be found online in the Supporting Information section.

## Supporting information


**Supporting Information 1** Table S1: Complete‐case analysis.

**Supporting Information 2** File S1: STROBE guideline for cross‐sectional studies.

## Data Availability

For this analysis, the researcher used the Demographic and Health Survey (DHS) dataset obtained from the DHS Program. To request the dataset for research purposes, a research proposal must be submitted to the DHS Program at https://dhsprogram.com/data/dataset_admin/index.cfm. The DHS Program typically reviews data requests within 24–48 h and notifies applicants upon approval. Once access is granted, the researcher can log in and download the requested data in the preferred format.
