# Supplementary material for: Updated Estimates of Childhood Diarrheal Morbidity in 11 Sub‐Saharan African Countries Using the Latest Demographic and Health Surveys (2021–2024): A Multilevel Modified Poisson Regression Analysis
Source: Biomed Res Int. 2026 Jul 13;2026:2495286. doi: 10.1155/bmri/2495286 (PMC13365792; doi:10.1155/bmri/2495286)
Supplement: Supplementary file 1 — Supporting Information 1 Table S1: Complete‐case analysis. [file BMRI-2026-2495286-s002.docx]

Supplementary Table S1 Complete-case analysis of factors associated with diarrheal morbidity among under-five children

| Variable | Category | Null model | Individual level | Community level | individual and community level |
| --- | --- | --- | --- | --- | --- |
|  |  |  | APR (95% CI) | APR (95% CI) | APR (95% CI) |
| Maternal age (in years) | 15-24 |  | 1 |  | 1 |
|  | 25–34 |  | 0.92 (0.88-0.96) |  | **0.9 (0.86-0.95)** |
|  | 35–49 |  | 0.84 (0.78-0.89) |  | **0.81 (0.76-0.86)** |
| Education | No education |  | 1 |  | 1 |
|  | Primary |  | 1.1 (1.06-1.17) |  | **1.15 (1.08-1.2)** |
|  | Secondary and above |  | 1.1 (1.03-1.17) |  | 1.04 (0.97-1.1) |
| Wealth index | Poor |  | 1 |  | 1 |
|  | Middle |  | 0.93 (0.88-0.99) |  | 0.95 (0.9-1.01) |
|  | Rich |  | 0.84 (0.8-0.89) |  | **0.85 (0.8-0.91)** |
| Marital status | Unmarried |  | 1 |  | 1 |
|  | Married |  | 1.1 (1.04-1.07) |  | 1.03 (0.97-1.1) |
| Employment status of mothers | Unemployed |  | 1 |  | 1 |
|  | Employed |  | 1.05 (1.01-1.1) |  | **1.03 (1.08-1.18)** |
| Religion | Muslim |  | 1 |  | 1 |
|  | Christian |  | 0.88 (0.84-0.93) |  | 1.05 (1.0-1.12) |
|  | Animist |  | 0.74 (0.67-0.82) |  | 1.02 (0.92-1.14) |
|  | No religion |  | 1.05 (0.98-1.13) |  | **1.14 (1.05-1.23)** |
| Residence | Urban |  |  | 1 | 1 |
|  | Rural |  |  | 0.96 (0.92-1.02) | **0.92 (0.86-0.99)** |
| Literacy | Illiterates |  | 1 |  | 1 |
|  | Literates |  | 0.87 (0.82-0.92) |  | **0.85 (0.82-0.89)** |
| Media exposure | Unexposed |  | 1 |  | 1 |
|  | Exposed |  | 1.12 (1.07-1.20) |  | **1.12 (1.08-1.16)** |
| Health insurance | No |  | 1 |  | 1 |
|  | Yes |  | 0.79 (0.72-0.87) |  | **0.83 (0.74-0.93)** |
| ANC visits | No ANC |  | 1 |  | 1 |
|  | 1–3 |  | 0.99 (0.92-1.07) |  | 1.02 (0.94-1.1) |
|  | 4 and above |  | 1.07 (0.99-1.15) |  | **1.09 (1.01-1.17)** |
| Postnatal checkup | No |  | 1 |  | 1 |
|  | Yes |  | 1.08 (1.03-1.13) |  | **1.1 (1.06-1.16)** |
| Health facility distance | No big problem |  |  | 1 | 1 |
|  | Big problem |  |  | 1.03 (0.99-1.08) | **1.05 (1.01-1.1)** |
| Child age (in months) | 0-36 |  | 1 |  | 1 |
|  | 37–59 |  | 0.41 (0.39-0.44) |  | **0.42 (0.4-0.44)** |
| Child sex | Males |  | 1 |  | 1 |
|  | Females |  | 0.93 (0.9-0.97) |  | **0.93 (0.9-0.97)** |
| Birth order | First |  | 1 |  | 1 |
|  | Subsequent |  | 0.92 (0.87-0.97) |  | **0.93 (0.88-0.93)** |
| Birth interval (in months) | ≤ 24 |  | 1 |  | 1 |
|  | > 24 |  | 0.93 (0.87-0.98) |  | **0.93 (0.88-0.99)** |
| Number of under-5 children | ≤ 2 |  | 1 |  | 1 |
|  | ≥ 3 |  | 1.15 (1.1-1.2) |  | 1.04 (0.99-1.09) |
| Water source | Unimproved |  | 1 |  | 1 |
|  | Improved |  | 1.0 (0.96-1.05) |  | 0.99 (0.94-1.05) |
| Toilet type | Unimproved |  | 1 |  | 1 |
|  | Improved |  | 1.1 (1.05-1.49) |  | 1.02 (0.97-1.07) |
| Underweight | No |  | 1 |  | 1 |
|  | Yes |  | 1.11 (1.03-1.20) |  | **1.1 (1.02-1.2)** |
| Stunted | No |  | 1 |  | 1 |
|  | Yes |  | 1.0 (0.94-1.06) |  | 1.0 (0.95-1.06) |
| Wasted | No |  | 1 |  | 1 |
|  | Yes |  | 1.16 (1.06-1.30) |  | **1.15 (1.05-1.25)** |
| Measles vaccine | No |  | 1 |  | 1 |
|  | Yes |  | 0.99 (0.94-1.04) |  | 0.97 (0.93-1.02) |
| Vitamin A | No |  | 1 |  | 1 |
|  | Yes |  | 1.13 (1.08-1.18) |  | **1.11 (1.06-1.16)** |
| Rotavirus | No |  | 1 |  | 1 |
|  | Yes |  | 1.19 (1.14-1.26) |  | **1.2 (1.14-1.26)** |
| Deworming | No |  | 1 |  | 1 |
|  | Yes |  | 1.13 (1.08-1.19) |  | **1.15 (1.09-1.2)** |
| Country | Burkina Faso |  |  | 1 | 1 |
|  | DR Congo |  |  | 0.95 (0.87-1.03) | 0.96(0.89-1.05) |
|  | Cote d'Ivoire |  |  | 0.63 (0.57-0.7) | **0.65 (0.58-0.72)** |
|  | Ghana |  |  | 0.7 (0.62-0.78) | **0.71 (0.6-0.85)** |
|  | Kenya |  |  | 0.92 (0.85-0.99) | **0.86 (0.79-0.93)** |
|  | Lesotho |  |  | 1.09 (0.95-1.25) | 0.97 (0.84-1.12) |
|  | Madagascar |  |  | 0.62 (0.56-0.68) | **0.59 (0.53-0.65)** |
|  | Mali |  |  | 1.14 (1.04-1.24) | **1.17 (1.06-1.28)** |
|  | Mozambique |  |  | 0.69 (0.61-0.77) | **0.67(0.59-0.76)** |
|  | Senegal |  |  | 1.28 (1.17-1.39) | **1.31 (1.19-1.45)** |
|  | Tanzania |  |  | 0.55 (0.5-0.61) | **0.52 (0.46-0.57)** |
| **Model fitness diagnosed** | |  |  |  |  |
| Device |  | -23414.15 | -23126.24 | -23152.41 | -22,933.14 |
| AIC |  | 46832.29 | 46316.47 | 46328.81 | 45950.27 |
| BIC |  | 46849.78 | 46596.18 | 46433.7 | 46317.39 |
| **Measure of variation** | |  |  |  |  |
| ICC |  | 1.06% | 0.85% | 0.89% | 0.8% |
| MRR |  | 1.2 | 1.17 | 1.18 | 1.17 |
| PCV |  | - | 19.84% | 16.55% | 24.5% |
